# Draft Genome Sequences of Four *Saccharibacter* sp. Strains Isolated from Native Bees

**DOI:** 10.1128/MRA.00022-20

**Published:** 2020-03-05

**Authors:** Eric A. Smith, Hoang Q. Vuong, Delaney L. Miller, Audrey J. Parish, Quinn S. McFrederick, Irene L. G. Newton

**Affiliations:** a Department of Biology, Indiana University, Bloomington, Indiana, USA; b Department of Entomology, University of California, Riverside, California, USA; University of Maryland School of Medicine

## Abstract

The genus Saccharibacter is currently understudied, with only one described species, Saccharibacter floricola, isolated from a flower. In an effort to better understand the microbes that come in contact with native bee pollinators, we isolated and sequenced four additional strains of *Saccharibacter* from native bees in the genera Melissodes and Anthophora. These genomes range in size from 2,104,494 to 2,316,791 bp (mean, 2,246,664 bp) and contain between 1,860 and 2,167 (mean, 2,060) protein-coding genes.

## ANNOUNCEMENT

The genus *Saccharibacter* currently comprises only one described species, isolated from the pollen of Japanese flowers (1). Saccharibacter floricola is an acetic acid bacterium (AAB). AAB have been shown to be associated with diverse insects that rely on sugar-rich diets (2, 3). As the genus *Saccharibacter* has been isolated from flowers, it may also come in contact with insect pollinators and play a role in their utilization of nectar as a food source. To determine whether this is the case and to bolster the available data for this potentially important genus, we isolated and sequenced the genomes of four *Saccharibacter* sp. strains isolated from native bee pollinators.

Four strains of *Saccharibacter* were isolated from native bees. Strains EH60, EH70, and E611 were isolated from bees in the genus *Anthophora*, and strain 17.LH.SD was isolated from a bee in the genus *Melissodes*, collected from Yosemite National Park, CA, and The Dalles, OR, respectively. Initial samples were streaked on MRS plus 2% fructose agar plates, after which single colonies were picked and grown in liquid culture. Strains were grown in yeast-peptone-glucose medium at 30°C with aeration. Total DNA was extracted using the DNeasy blood and tissue kit (Qiagen, Hilden, Germany), and sequencing library preparation was performed using the NEBNext Ultra II DNA library preparation kit (NEB, Ipswich, MA), both according to the manufacturers’ protocols. The resulting libraries were subjected to 250-bp paired-end sequencing on the Illumina NextSeq 500 platform (version 2 chemistry) (strain 17.LH.SD was sequenced on the Illumina MiSeq platform [version 2 chemistry] with 300-bp paired-end sequencing) at the Indiana University Center for Genomics and Bioinformatics (Bloomington, IN). These sequencing runs generated 104,267 to 941,404 (mean, 551,094) read pairs.

Initial *de novo* assembly of these strains was performed using MaSuRCA version 3.2.8 with default settings (4). Reads were not subjected to quality control (QC) prior to assembly, as MaSuRCA performs internal QC during the assembly process. Additionally, reads were randomly subsampled down to approximately 50× coverage prior to assembly (100× for strain EH70). The completeness of the assemblies was assessed using both BUSCO (version 3; alphaproteobacteria lineage data set) (5) and CheckM (version 1.1.1) (6). Both of these tools indicated that the assemblies were >99% complete.

The assemblies resulted in 13 to 30 (mean, 23) contigs comprising 2,104,494 to 2,316,791 bp (mean, 2,246,664 bp), with an *N*_50_ contig length of 297,436 to 461,108 bp (mean, 341,556 bp). The GC content of these strains was 48.61 to 51.47% (mean, 50.56%). Annotation was carried out with NCBI’s Prokaryotic Genome Annotation Pipeline (PGAP) (7), which predicted between 1,860 and 2,167 (mean, 2,060) protein-coding genes and 1 to 4 (mean, 2) rRNAs. Assembly statistics are summarized in Table 1.

**TABLE 1:** Relevant assembly information and statistics

Strain	GenBank accession no.	No. of contigs	Genome size (Mb)	% GC content	*N*_50_ (bp)	*L*_50_	Host genus	Isolation location^*a*^
EH60	WVHQ00000000	30	2.32	51.47	301,953	4	Anthophora	Yosemite NP, CA
EH611	WVHP00000000	25	2.28	51.47	303,728	4	*Anthophora*	Yosemite NP, CA
EH70	WVHN00000000	26	2.29	50.70	297,436	4	*Anthophora*	Yosemite NP, CA
17.LH.SD	WVHO00000000	13	2.10	48.61	461,108	2	Melissodes	The Dalles, OR

a NP, National Park.

### Data availability.

These whole-genome shotgun projects have been deposited at DDBJ/ENA/GenBank under the accession numbers WVHN00000000, WVHO00000000, WVHP00000000, and WVHQ00000000. The versions described in this paper are versions WVHN01000000, WVHO01000000, WVHP01000000, and WVHQ01000000. Sequencing reads have been deposited in the Sequence Read Archive (SRA) under the accession numbers SRR10728926 to SRR10728929.
